# Perfectionism and Adolescent Athletes’ Burnout: The Serial Mediation of Motivation and Coping Style

**DOI:** 10.3390/bs14111011

**Published:** 2024-10-31

**Authors:** Aiai Xu, Xiaobing Luo, Xueqing Qiu, Changfen Lu

**Affiliations:** 1 School of Physical Education and Sports, Central China Normal University, Wuhan 430079, China; xaa@mails.ccnu.edu.cn (A.X.); lxbccnu@126.com (X.L.); qiuxq1229@163.com (X.Q.); 2 Nanyuanfang Primary School, Wuhan 430035, China

**Keywords:** perfectionistic strivings, perfectionistic concerns, burnout, intrinsic motivation, amotivation, problem-focused coping, emotion-focused coping

## Abstract

Preventing and reducing adolescent athletes’ risk of burnout can help promote long-term sports participation, improve performance, and maintain psychological well-being. The present study examined the associations between perfectionism and burnout among Chinese adolescent athletes and the mediating role of motivation and coping styles. A total of 243 Chinese adolescent athletes (78% boys; Mage = 17.8; SD = 2.62) completed the Sport Multidimensional Perfectionism Scale for China, the Situational Motivation Scale, the Coping Scale for Chinese Athletes, and the Athlete Burnout Questionnaire to assess perfectionism (strivings and concerns), coping styles (problem-focused and emotion-focused), motivation (intrinsic and amotivation), and burnout. Path analyses indicated that intrinsic motivation and coping styles (problem-focused and emotion-focused) serially mediated the relationship between perfectionistic strivings and burnout. Problem-focused coping mediated the relationship between perfectionistic concerns and burnout. These findings contribute to a model of the effect of perfectionism on adolescent athletes’ burnout, provide support for the self-determination theory, and suggest a feasible approach for mitigating burnout in this group.

## 1. Introduction

Athlete burnout is a syndrome characterized by three core symptoms: emotional and physical exhaustion, perceptions of reduced achievement, and sport devaluation [1]. A cross-temporal meta-analysis suggested that burnout symptoms have increased over the past two decades [2]. Although, the prevalence of burnout was influenced by the measurement tools and sample representativeness [3]. Athletes’ burnout is associated with many negative outcomes including reduced performance and sport dropout [4]. Prevention of this problem depends on identifying factors that predict athletes’ burnout and the mechanisms of these effects. Perfectionism has been acknowledged as a robust antecedent of athletes’ burnout [5]. According to self-determination theory, athletes’ perfectionism may be related to their motivation. If athletes’ perfectionism is due to external pressures or to fulfill the expectations of others, this may lead to a higher risk of burnout [6]. However, researchers have mostly focused on the role of autonomous and controlled motivation in the relationship. Intrinsic motivation and amotivation, as two extremes of the motivation continuum, better capture changes in burnout [7]. Moreover, intrinsic motivation as compared to amotivation is assumed to lead to more flexible and positive stress appraisals [8] and hence to higher levels of coping skills [9]. Therefore, the purpose of this study was to investigate the relationship between perfectionism and burnout in adolescent athletes and the serial mediating roles of motivation and coping styles.

### 1.1. Perfectionism and Burnout

Perfectionism is a personality disposition characterized by striving for flawlessness and setting exceedingly high standards for performance, accompanied by overly critical self-evaluations [10,11,12]. Perfectionism is multidimensional and includes six dimensions: personal standards, concern over mistakes, perceived parental pressure, perceived coach pressure, doubts about actions, and organization [13]. Based on this, some researchers used factor analysis to extract two-dimensional perfectionism with high aggregation from different scales: perfectionistic strivings and perfectionistic concerns [14]. For example, personal standards and organization are included in perfectionistic strivings; concerns over mistakes, perceived parental pressure, perceived coach pressure, and doubts about actions are included in perfectionistic concerns [15]. While perfectionistic strivings refers to setting high standards and goals for oneself, perfectionistic concerns refers to when individuals impose unrealistic standards and expectations on themselves, resulting in overly harsh evaluations of their own behavior. This represents two aspects of perfectionism: adaptive and non-adaptive. The adaptive aspect of perfectionism is seen in self-directed excellence, high-performance standards, positive mood, and excellent performance; in contrast, the maladaptive aspect of perfectionism is evident in perceived criticism and self-doubt, and it is associated with negative emotions such as anxiety and restlessness [16].

Perfectionistic strivings and perfectionistic concerns were inconsistently associated with burnout. According to the self-determination theory, the satisfaction of the three basic psychological needs of autonomy, competence, and relationships enhances the individual’s internal motivation and promotes the internalization of external motivation [6]. If athletes pursue perfection because of intrinsic interest and fulfillment, this may promote their mental health and performance. However, if athletes strive for perfectionism due to external pressures or to meet the expectations of others, they may lose sight of these basic needs by focusing too much on achievement and external standards, leading to burnout. Two longitudinal studies of adolescent athletes have demonstrated a negative or non-significant association between perfectionistic strivings and burnout [17,18]. There is a positive relationship between perfectionistic concerns and burnout [19]. A meta-analysis found that perfectionistic strivings has a small negative correlation with overall burnout, while perfectionistic concerns have a moderate to strong positive correlation with burnout [20]. According to Hill [21], motivation is crucial to understanding these relationships. Identifying the motivating mechanisms triggered by perfectionism may have implications for preventing or decreasing athletes’ burnout. On this basis, it was proposed in this study that perfectionistic strivings will be negatively associated with athlete burnout (H1a), and perfectionistic concerns will be positively associated with athlete burnout (H1b).

### 1.2. Perfectionism, Motivation, and Burnout

In the explanatory framework of self-determination theory, motivation plays a crucial role in the relationship between perfectionism and burnout [22]. According to the theory, human motivation is a dynamic continuum that can be divided into categories representing different types of motivation. Intrinsic motivation, extrinsic motivation, and amotivation vary based on the degree of self-determination. Intrinsic motivation is considered the most self-determined form, as it originates from an individual’s interests and intrinsic sense of accomplishment. Extrinsic motivation is contingent upon attaining or avoiding specific outcomes. Amotivation represents the least self-determined form of motivation, as it is characterized by an individual’s inability to perceive the connection between their actions and outcomes [23].

Research suggests that amotivation is the motivation type with the strongest relationship associated with burnout [24,25]. Intrinsic motivation and amotivation are situated at opposite ends of the motivation continuum and show the maximal difference in self-determination; they may be most likely to capture the motivational characteristics associated with burnout [7]. A systematic review suggested that intrinsic motivation is negatively correlated with burnout and amotivation is positively correlated with burnout [26]. A subsequent study provided further evidence for this conclusion: the relationship between perfectionism and burnout was mediated by the two forms of motivation [7]. Consistent with this finding, in a study of 185 athletes, maladaptive perfectionism was associated with higher amotivation in athletes [27]. A study of 487 swimmers also showed adaptive perfectionism negatively predicted amotivation [28]. On this basis, it was proposed in this study that motivation will mediate the association between perfectionism and burnout (H2).

### 1.3. Perfectionism, Coping Style, and Burnout

Coping refers to an individual’s behavioral and cognitive endeavors to manage stressful situations’ demands effectively [29]. Coping styles are most described as either problem-focused or emotion-focused [30,31]. The former pertains to an individual’s subjective assessment of a stressful situation and their attempts to actively seek ways to modify it. The latter pertains to an individual’s attempt to manage the anxiety induced by the stressful situation. In sports, perfectionism is considered a potential antecedent of effective coping [32], and adaptive perfectionism has been shown to positively influence athletes’ coping strategies [33]. Shin et al. [31] conducted a meta-analysis of 36 correlational studies of the association between various coping strategies and three dimensions of burnout symptoms. The result showed that problem-focused coping was negatively associated with the three dimensions of burnout, whereas emotion-focused coping was positively associated with the three dimensions. The mediating role of coping style in the association between perfectionism and burnout has been confirmed in previous studies; however, these investigations were conducted outside of the sports domain [34,35]. In contrast, a study of 173 American intercollegiate varsity athletes showed the mediating role of coping style in the association between perfectionism and burnout in the sports setting did not yield statistically significant results [36]. Therefore, this study aims to test the mediating role of coping style in the relationship between perfectionism and burnout. On this basis, it was proposed in this study that coping styles will mediate the association between perfectionism and adolescent athlete burnout (H3).

### 1.4. Perfectionism, Motivation, Coping Style, and Burnout

The correlations between perfectionism and burnout may be attributed to the complex associations between the type of motivation and the type of coping style elicited by the motivation [37]. In the sports domain, a study of athletes aged 14–18 documented a positive correlation between individuals’ motivation to participate in sports and their situational coping strategies during sports competitions [38]. Individuals who exhibit higher autonomous motivation have been shown to make a more positive and rapid appraisal of stress than those with controlled motivation [8]. In a study of 211 junior athletes, high autonomous motivation was associated with positive coping styles, and conversely, high controlled motivation was associated with emotional coping styles [39]. Individuals with high self-determination may interpret the difficulties they encounter as challenges rather than threats, thus promoting problem-focused coping styles. It has been suggested that autonomous motivation mediates the relationship between perfectionistic strivings and adaptive coping [32]. Mouratidis et al. [9] have argued that motivation can explain the relationship between perfectionism and coping. Thus, it appears that perfectionism may be associated with burnout in adolescent athletes through their motivation and coping styles. On this basis, it was proposed in this study that the association between perfectionism and adolescent athlete burnout will be serially mediated by motivation and coping styles (H4).

## 2. Materials and Methods

### 2.1. Participants

A sample of 330 Chinese student-athletes was recruited online for this study. They were from sports schools, universities, and provincial and national teams. Informed consent was obtained from school principals and parents for their children’s participation before they completed the questionnaire. In the group of 330 participants, 250 met the World Health Organization’s definition of adolescence which is the period of life from childhood to adulthood between the ages of 10 and 19 (World Health Organization, 2020). The ultimate sample consisted of 250 adolescent athletes (196 boys and 54 girls; 63.8% in high school; 54.3% from rural areas) aged 12 to 19 (M = 17.80, SD = 2.62) from various sports (61 in athletics, 41 in basketball, 32 in volleyball, 29 in football, 15 in tennis, and 71 in other sports [e.g., shooting; Taekwondo]). They varied in the number of years in training (M = 4.5; SD = 3.16) and competition level (national level 3 and above). This study was approved by the Ethics Committee of Central China Normal University.

### 2.2. Instruments

#### 2.2.1. Athlete Burnout

Athlete burnout was measured with the Athlete Burnout Questionnaire (ABQ) [1] using the Chinese version developed by Zhang and Mao [40]. The ABQ has three dimensions, including reduced sense of accomplishment (5 items, e.g., “I didn’t achieve better results”), emotional and physical exhaustion (5 items, e.g., “I felt extremely fatigued during training and competitions”), and devaluation (5 items, e.g., “I don’t care about athletic performance as much as I used to”). A five-point Likert scale ranging from 1 (never) to 5 (always) was used. Some items were scored in reverse, and all items were summed and averaged for a scale score reflecting global burnout; higher scores represented higher levels of burnout. A study using the Chinese version of the ABQ with an excellent athlete (Mage = 19.88) population reported satisfactory internal consistency (Cronbach’s α = 0.79) [41].

#### 2.2.2. Perfectionism

Perfectionism was measured using the Sport Multidimensional Perfectionism Scale for China (MPS-S-C) [42], which was adapted from the Sport Multidimensional Perfectionism Scale (MPS-S) [43]. Lian et al. [42] translated and analyzed the factors to make the scale fit the Chinese cultural background. The MPS-S-C has five subscales: personal standards (9 items), rumination (7 items), concern over mistakes (7 items), perceived parental pressure (7 items), and perceived coaching pressure (7 items). We used personal standards as the indicator of perfectionistic strivings and the other four subscales as indicators of perfectionistic concerns [44]. A five-point Likert scale ranged from 1 (disagree) to 5 (agree). All items were summed and averaged for a scale score reflecting perfectionism; higher scores represented higher levels of perfectionism. The MPS-S-C has demonstrated adequate internal consistency in a sample of Chinese athletes (Cronbach’s α = 0.83) [42].

#### 2.2.3. Motivation

Motivation about sports was measured with the Situational Motivation Scale (SIMS) [45], using the Chinese version developed by Zhang and Mao [40]. The scale has four dimensions: intrinsic motivation (4 items), amotivation (4 items), identification principle (4 items), and externalization principle (4 items). Two of the four subscales were used in the current study: intrinsic motivation (e.g., “Because I find this sport interesting”) and amotivation (e.g., “There may be good reasons to do this activity, but I do not see any”). Items were rated using a five-point Likert scale from 1 (not at all in agreement) to 5 (entirely in agreement). Some items were scored in reverse, and the average score of each of the 4-item scales was used for adolescent athletes’ intrinsic motivation and amotivation. The Chinese version of the Situational Motivation Scale showed good internal consistency in a sample of Chinese college athletes (intrinsic motivation’s Cronbach’s α = 0.83, amotivation’s Cronbach’s α = 0.58) [40].

#### 2.2.4. Coping Styles

Coping styles were measured using the Coping Scale for Chinese Athletes (CSCA) [46]. The CSCA has four dimensions: problem-focused coping (6 items), emotion-focused coping (6 items), avoidance coping (6 items), and transcendence coping (6 items). Two of the four subscales were used in the current study: problem-focused coping (e.g., “Solve the problem one step at a time”) and emotion-focused coping (e.g., “Try to calm yourself down”). The items were rated using a five-point Likert scale ranging from 1 (never) to 5 (always). The average score of each of the 6-item scales was used for adolescent athletes’ problem-focused coping and emotion-focused coping. The problem-focused coping and emotion-focused coping subscales have both shown good internal consistency in a Chinese athlete sample (Cronbach’s α = 0.72 and 0.77) [46].

### 2.3. Analytic Strategy

First, following Tabachnick and Fidell [47], we checked for missing values, outliers, and normality, and assessed common method bias, multicollinearity, and each measure’s internal consistency (Cronbach’s α and Corrected Item-Total Correlation, CITC). Following these preliminary analyses, descriptive statistics and Pearson correlations were generated to examine the relationships among perfectionism, motivation, coping, and burnout. Finally, the PROCESS macro for SPSS 24.0 [48] was used to test the hypotheses.

## 3. Results

### 3.1. Preliminary Analyses

There were no missing data. Next, we screened for univariate outliers; after removing seven participants (Mahalanobis distance > critical value at *p* < 0.001) [47], a total of 243 participants remained. Given a power at 0.80 and a significance level at 0.05 for a medium effect (*f*^2^ = 0.15; R^2^ = 0.13) [49] with respect to four predictors in multiple regression, at least 85 participants are needed. The number of participants in this study was sufficient. The skewness and kurtosis of all variables were less than 2, indicating no substantial deviation from the normal distribution (see Table 1). Cronbach’s α of the amotivation subscale was less than 0.6, so the second item with a CITC value less than 0.4 was dropped and three items remained. Thus, Cronbach’s α of all the scales was greater than 0.7, indicating that the scales had good internal consistency (see Table 1). Harman’s single-factor test showed that the total variance extracted by one factor was 16.16%. The multicollinearity test showed that the VIF ranged between 1.10 and 2.54. Therefore, there was no substantial common method bias or multicollinearity.

### 3.2. Descriptive Statistics and Correlation Analysis

The descriptive statistics and bivariate correlations are reported in Table 2. Participants reported moderate-to-high perfectionistic strivings and concerns, intrinsic motivation, and coping styles, and moderate levels of athlete burnout. In addition, participants reported low-to-moderate amotivation. The correlation analyses showed that perfectionistic strivings was significantly negatively correlated with burnout, and perfectionistic concerns was significantly positively correlated with burnout. The two perfectionisms were significantly positively correlated with intrinsic motivation and the two coping styles and significantly negatively correlated with amotivation and athlete burnout. Intrinsic motivation was significantly positively correlated with the two coping styles and significantly negatively correlated with athlete burnout. Amotivation was significantly negatively correlated with the two coping styles and significantly positively correlated with athlete burnout. The two coping styles were significantly negatively correlated with athlete burnout.

### 3.3. Pathway Tests

#### 3.3.1. Perfectionistic Strivings and Burnout

**Figure 1:**
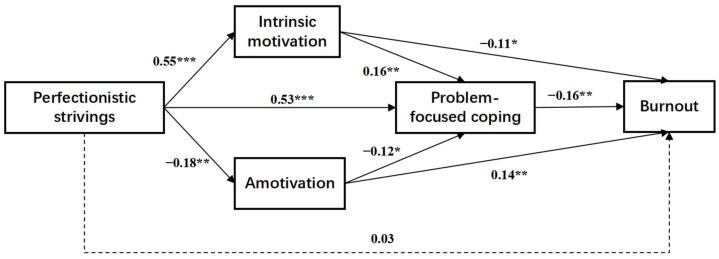
Motivation and problem-focused coping as mediators in the relationship between perfectionistic strivings and adolescent athlete burnout (Model 1). Notes: *n* = 243. * *p* < 0.05, ** *p* < 0.01, and *** *p* < 0.001. The numbers in Figure 1 are unstandardized coefficients. Solid lines represent significant paths, while the dashed line represents a nonsignificant path.

**Figure 2:**
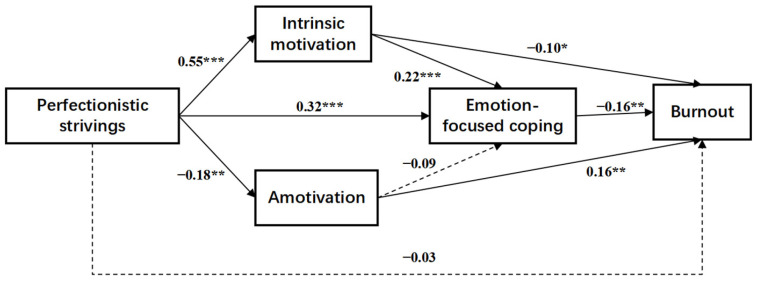
Motivation and emotion-focused coping as mediators in the relationship between perfectionistic strivings and adolescent athlete burnout (Model 2). Notes: *n* = 243. * *p* < 0.05, ** *p* < 0.01, and *** *p* < 0.001. The numbers in Figure 2 are unstandardized coefficients. Solid lines represent significant paths, while dashed lines represent nonsignificant paths.

Template 80 in the PROCESS macro for SPSS [48] was used to explore the role of motivation and coping styles in the relationship between perfectionistic strivings and burnout. We tested mediation twice, once for problem-focused coping (Model 1, illustrated in Figure 1) and once for emotion-focused coping (Model 2, illustrated in Figure 2).

The pathway test results are shown in Table 3. The total effect of perfectionistic strivings on burnout was significant (effect = 0.156; 95% CI = −0.252 to −0.060). The direct effect of perfectionistic strivings on burnout was nonsignificant (effect = 0.029; 95% CI = −0.091 to 0.149), but the total indirect effect was significant (effect = −0.185; 95% CI = −0.281 to −0.098). Perfectionistic strivings had negative indirect effects on athlete burnout via intrinsic motivation (effect = −0.060; 95% CI = −0.112 to −0.016), amotivation (effect = −0.025; 95% CI = −0.051 to −0.004), and problem-focused coping (effect = −0.083; 95% CI = −0.153 to −0.024). In addition, perfectionistic strivings significantly and negatively predicted athlete burnout through a serial mediation of intrinsic motivation and problem-focused coping (effect = −0.014; 95% CI = −0.030 to −0.002) and through a serial mediation of amotivation and problem-focused coping (effect = −0.003; 95% CI = −0.010 to 0.000).

The pathway test analyses revealed the roles of motivation and emotion-focused coping in the association between perfectionistic strivings and athlete burnout (Table 4). The total effect of perfectionistic strivings on burnout was significant (effect = 0.156; 95% CI = −0.252 to −0.060). The direct effect of perfectionistic strivings on burnout was not significant (effect = −0.004; 95% CI = −0.112 to 0.105), but the total indirect effect was significant (effect = −0.152; 95% CI = −0.226 to −0.086). Perfectionistic strivings had negative indirect effects on athlete burnout via intrinsic motivation (effect = −0.056; 95% CI = −0.112 to −0.011), amotivation (effect = −0.028; 95% CI = −0.055 to −0.005), and emotion-focused coping (effect = −0.050; 95% CI = −0.090 to −0.017). In addition, perfectionistic strivings significantly and negatively predicted athlete burnout through a serial mediation of intrinsic motivation and emotion-focused coping (effect = −0.019; 95% CI = −0.037 to −0.005). The serial mediation of amotivation and emotion-focused coping between perfectionistic strivings and burnout was not significant (effect = −0.003; 95% CI = −0.005 to 0.006).

#### 3.3.2. Perfectionistic Concerns and Burnout

**Figure 3:**
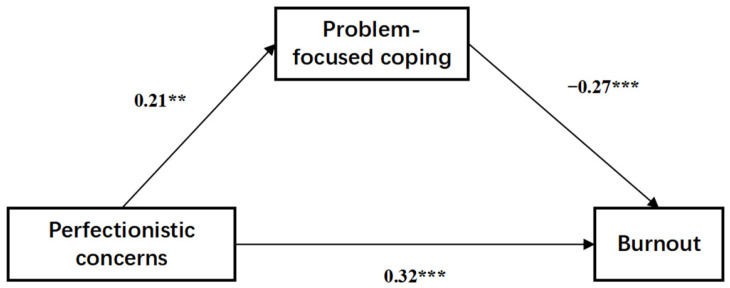
Problem-focused coping as a mediator in the relationship between perfectionistic concerns and adolescent athlete burnout (Model 3). Notes: *n* = 243. ** *p* < 0.01, and *** *p* < 0.001. The numbers in Figure 3 are unstandardized coefficients. Solid lines represent significant paths.

According to the results of the correlation analysis, motivation was not significantly correlated with the other three variables, so it was not possible to test the serial mediating effect of motivation and coping styles between perfectionistic concerns and burnout. Instead, there was a two-by-two correlation between perfectionistic concerns, problem-focused coping, and burnout. Template 4 in the PROCESS macro for SPSS [48] was used to explore the role of problem-focused coping styles in the relationship between perfectionistic concerns and burnout (Figure 3).

The pathway test results are shown in Table 5. Perfectionistic concerns had a positive total effect on burnout (effect = 0.322; 95% CI = 0.197 to 0.448). The direct effect of perfectionistic concerns on burnout was significant (effect = 0.379; 95% CI = 0.261 to 0.497). Perfectionistic concerns had a negative indirect effect on athlete burnout via problem-focused coping (effect = −0.056; 95% CI = −0.113 to −0.008).

## 4. Discussion

This cross-sectional study examined the relationship between perfectionism and burnout in a sample of 243 Chinese adolescent athletes. Tests of mediation illuminated three mechanisms by which perfectionistic strivings is associated with athletes’ burnout. Two of these mechanisms involved motivation (intrinsic motivation and amotivation) and coping style (problem-focused and emotion-focused coping style) as a single mediator in support of the hypotheses. Also in support of the hypotheses was a serial mediating effect of intrinsic motivation and coping style (problem-focused and emotion-focused coping style). In addition, problem-focused coping mediated the relationship between perfectionistic concerns and burnout. The results have implications for preventing and reducing adolescent athletes’ burnout by enhancing their intrinsic motivation and adopting coping strategies that are effective in a given situation.

### 4.1. The Relationship Between Perfectionism and Burnout

The results indicated that perfectionistic strivings was significantly negatively correlated with burnout, while perfectionistic concerns was significantly positively correlated with burnout, confirming hypotheses H1a and H1b. This is consistent with previous research, which found opposing relationships between the two dimensions of perfectionism and burnout [39]. A longitudinal study on junior athletes showed that perfectionistic strivings predicted a decrease in athletes’ burnout, while perfectionistic concerns were a risk factor for increased burnout [17]. Perfectionistic strivers may experience satisfaction and motivation from their pursuit of achievement, thereby reducing burnout [50]. In contrast, perfectionistic concerns may feel pressure and frustration from their inability to meet expectations, leading to burnout [51]. In practice, cognitive–behavioral therapy can help athletes identify and improve maladaptive beliefs that contribute to perfectionistic thinking and behavior [52]. Through this therapy, athletes can set more realistic goals, reduce fear of failure, and develop healthier self-evaluation methods.

### 4.2. The Mediation Role of Motivation

The research results indicate that intrinsic motivation mediates the relationship between perfectionistic strivings and burnout, which supports H2. This finding is consistent with previous studies [7]. Perfectionistic strivings is positively correlated with self-determined motivation and intrinsic motivation for achievement [53], meaning that perfectionists exhibit greater drive when facing high standards and challenges. Additionally, athletes with perfectionistic strivings are more likely to act out of intrinsic interest and satisfaction rather than external pressure or control. According to self-determination theory, athletes with strong intrinsic motivation have needs for autonomy, competence, and relatedness; the satisfaction of these basic psychological needs results in lower levels of burnout [23]. Furthermore, the results also indicate that amotivation mediates the relationship between perfectionistic strivings and burnout, further supporting H2. Specifically, perfectionists may experience ongoing self-criticism and negative emotions in their pursuit of high standards, which can weaken their intrinsic and extrinsic motivation, ultimately leading to a state of amotivation [54]. This state of amotivation further diminishes athletes’ interest in and willingness to engage in activities, increasing the risk of burnout.

The research results did not find a mediating role of intrinsic motivation and amotivation between perfectionism concern and burnout, which is inconsistent with previous findings. A study suggested that perfectionism concern may predict amotivation, as this dimension of perfectionism includes a sense of helplessness when striving to meet externally imposed standards [55]. For example, a study indicated that amotivation mediates the relationship between perfectionism concern and burnout [56]. The inconsistency of this study’s results with previous ones may be due to other types of motivation (such as extrinsic motivation) playing a role between perfectionism concern and burnout. For instance, research has shown that perfectionism concern positively influences burnout through controlled motivation [39]. Therefore, future studies could explore the relationships between various types of motivation and different forms of perfectionism.

### 4.3. The Mediation Role of Coping Styles

The research results indicated that both problem-focused coping and emotion-focused coping mediate the relationship between perfectionistic strivings and burnout, supporting hypothesis H3. Based on the cognitive–affective model of burnout, problem-focused coping may lead to lower levels of burnout by reducing the frequency and duration of stress [37]. For example, problem-focused coping has been found to alleviate burnout [57]. Emotion-focused coping emphasizes regulating emotional discomfort caused by stress. This strategy can help individuals mitigate the negative emotions arising from perfectionistic strivings, thereby indirectly reducing the occurrence of burnout. Although previous research suggested that the directional impact of emotion-focused coping is ambiguous [58], one study has shown that problem-focused coping is significantly negatively correlated with emotional and physical exhaustion and perceptions of reduced achievement in burnout symptoms, while emotion-focused coping is significantly negatively correlated with perceptions of reduced achievement [59]. This supports the findings of the current study regarding the role of both coping strategies in reducing burnout. Additionally, the results indicate that problem-focused coping mediates the relationship between perfectionistic concerns and burnout. The negative impacts of perfectionistic concerns may be offset by athletes engaging in problem-focused coping [14].

### 4.4. The Serial Mediation Role of Motivation and Coping Style

This study also found that perfectionistic strivings was associated with burnout through a serial mediation effect involving intrinsic motivation and the two coping styles, supporting hypothesis H4. Previous research has shown that motivation and coping strategies can mitigate symptoms of burnout [19]. This study not only validates this conclusion but also expands upon it, demonstrating that problem-focused coping and motivation can mediate their relationship in parallel as well as in a sequential manner. Athletes with perfectionistic strivings pursue goals that align with their personal standards and values, which can enhance intrinsic motivation. The increase in intrinsic motivation leads them to adopt proactive strategies to solve problems to meet their high standards. Both problem-focused coping and emotion-focused coping are considered task-oriented coping strategies [32]. When individuals employ task-oriented coping strategies, they may feel a greater sense of control over stressors, thereby reducing burnout. Therefore, in practice, sports psychologists and coaches can help athletes develop task-oriented coping strategies to reduce the burnout that may result from their perfectionistic tendencies.

However, the present study did not find significant serial mediation effects of amotivation and coping style on the relationship between perfectionistic strivings and burnout, an important observation to highlight. The findings indicated that both coping styles can contribute to alleviating burnout. However, amotivation may pose challenges in effectively mobilizing resources to cope with stressful conditions. People who lack motivation tend to adopt a negative coping style such as avoidance or no coping style. Additional research is warranted to explore the coping strategies employed by adolescent athletes who exhibit a lack of motivation, as well as their potential risk of burnout.

### 4.5. Limitations and Prospects

This study has several limitations. First and foremost, it is imperative to acknowledge that the present cross-sectional survey study lacks evidence to demonstrate a causal association. Mediation based on cross-sectional designs increases the probability of obtaining biased parameter estimates [60]. Burnout is known to arise during extended periods of stress, and future studies should incorporate a time series to investigate the predictors of burnout and changes in burnout over time. Secondly, variables such as gender and type of sport may affect the level of burnout in athletes; so in the future, more control variables should be considered to test the relationship between perfectionism and burnout. Next, although this study used adolescent athletes as participants, it did not establish a sports context. Subsequent research could incorporate a sports context to enhance athletes’ motivation and coping abilities during sports scenarios. Finally, the perfectionism 2 × 2 model theory has been demonstrated [15], and this study did not consider the interaction of the two types of perfectionism from an individual difference perspective. The relationship between perfectionism and burnout will be explored in the future on this basis.

## 5. Conclusions

This study analyzed cross-sectional data from 243 adolescent athletes to explore the relationships among perfectionism, motivation, coping styles, and burnout. The findings indicated that perfectionistic strivings was associated with burnout not only through separate mediators of motivation (intrinsic motivation and amotivation) and coping styles (problem-focused coping and emotion-focused coping) but also through a serial mediation of intrinsic motivation and coping styles. Perfectionistic concerns was associated with burnout through the mediating role of problem-focused coping. This research supports self-determination theory and offers theoretical insights and practical pathways to help athletes reduce burnout.

## Figures and Tables

**Table 1:** Skewness, Kurtosis, and Cronbach’s α of all variables.

Variable	Skewness	Kurtosis	Cronbach’s α
Athlete burnout	0.47	0.46	0.86
Perfectionistic strivings	−0.39	0.53	0.88
Perfectionistic concerns	0.05	−0.43	0.87
Intrinsic motivation	−0.79	0.30	0.88
Amotivation (without an item)	−0.04	−0.45	0.76
Problem-focused coping	−0.30	0.14	0.87
Emotion-focused coping	−0.56	0.32	0.87

Note: *n* = 243.

**Table 2:** Descriptive statistics and bivariate correlations among research variables.

Variable	M ± SD	1	2	3	4	5	6	7
1. Athlete burnout	2.34 ± 0.58	-						
2. Perfectionistic strivings	3.82 ± 0.75	−0.201 *	-					
3. Perfectionistic concerns	3.12 ± 0.56	0.310 **	0.495 **	-				
4. Intrinsic motivation	4.02 ± 0.90	−0.272 **	0.462 **	0.110	-			
5. Amotivation	2.06 ± 0.85	0.331 **	−0.305 **	−0.010	−0.363 **	-		
6. Problem-focused coping	3.76 ± 0.79	−0.305 **	0.607 **	0.149 *	0.433 **	−0.353 **	-	
7. Emotion-focused coping	3.81 ± 0.79	−0.293 **	0.425 **	0.092	0.392 **	−0.243 **	0.643 **	-

Note: *n* = 243, M ± SD = Mean ± Standard Deviation, ** *p* < 0.01, * *p* < 0.05.

**Table 3:** Decomposed effects, standard errors, and 95% confidence intervals for the effect of perfectionistic strivings, motivation, and problem-focused coping on athlete burnout.

Pathways	Effect	SE	95%CI (LL, UL)
1 Total effect	−0.156	0.049	(−0.252, −0.060)
2 Direct effects	0.029	0.061	(−0.091, 0.149)
3 Total indirect effects	−0.185	0.047	(−0.281, −0.098)
4 Perfectionistic strivings → intrinsic motivation → athlete burnout	−0.060	0.024	(−0.112, −0.016)
5 Perfectionistic strivings → amotivation → athlete burnout	−0.025	0.012	(−0.051, −0.004)
6 Perfectionistic strivings → problem-focused coping → athlete burnout	−0.083	0.033	(−0.153, −0.024)
7 Perfectionistic strivings → intrinsic motivation → problem-focused coping → athlete burnout	−0.014	0.007	(−0.030, −0.002)
8 Perfectionistic strivings → amotivation → problem-focused coping → athlete burnout	−0.003	0.003	(−0.010, 0.000)

Note: *n* = 243, SE = Standard Error, CI = Confidence Interval, LL = Lower Limit, and UL = Upper Limit.

**Table 4:** Decomposed effects, standard errors, and 95% confidence intervals for the effect of perfectionistic strivings, motivation, and emotion-focused coping on athlete burnout.

Pathways	Effect	SE	95%CI (LL, UL)
1 Total effect	−0.156	0.049	(−0.252, −0.060)
2 Direct effects	−0.004	0.055	(−0.112, 0.105)
3 Total indirect effects	−0.152	0.035	(−0.226, −0.086)
4 Perfectionistic strivings → intrinsic motivation → athlete burnout	−0.056	0.026	(−0.112, −0.011)
5 Perfectionistic strivings → amotivation → athlete burnout	−0.028	0.013	(−0.055, −0.005)
6 Perfectionistic strivings → emotion-focused coping → athlete burnout	−0.050	0.019	(−0.090, −0.017)
7 Perfectionistic strivings → intrinsic motivation → emotion-focused coping → athlete burnout	−0.019	0.008	(−0.037, −0.005)
8 Perfectionistic strivings → amotivation → emotion-focused coping → athlete burnout	−0.003	0.002	(−0.005, 0.006)

Note: *n* = 243, SE = Standard Error, CI = Confidence Interval, LL = Lower Limit, and UL = Upper Limit.

**Table 5:** Decomposed effects, standard errors, and 95% confidence intervals for the effect of perfectionistic concerns and problem-focused coping on athlete burnout.

Pathways	Effect	SE	95%CI (LL, UL)
1 Total effect	0.322	0.064	(0.197, 0.448)
2 Direct effects	0.379	0.060	(0.261, 0.497)
3 Perfectionistic concerns → problem-focused coping → athlete burnout	−0.056	0.027	(−0.113, −0.008)

Note: *n* = 243, SE = Standard Error, CI = Confidence Interval, LL = Lower Limit, and UL = Upper Limit.

## Data Availability

The anonymized data that support the findings of this study are available on request from the corresponding author. The data are not publicly available due to containing information that may comprise the participants’ privacy.

## References

[1] Raedeke T.D., Smith A.L. (2001). Development and Preliminary Validation of an Athlete Burnout Measure. J. Sport Exerc. Psychol..

[2] Madigan D.J., Olsson L.F., Hill A.P., Curran T. (2022). Athlete Burnout Symptoms Are Increasing: A Cross-Temporal Meta-Analysis of Average Levels from 1997 to 2019. J. Sport Exerc. Psychol..

[3] Tenenbaum G., Eklund R.C., Boiangin N. (2020). Handbook of Sport Psychology.

[4] Isoard-Gautheur S., Guillet-Descas E., Gustafsson H. (2016). Athlete Burnout and the Risk of Dropout Among Young Elite Handball Players. Sport Psychol..

[5] Gould D., Dieffenbach K., Moffett A. (2002). Psychological Characteristics and Their Development in Olympic Champions. J. Appl. Sport Psychol..

[6] Deci E.L., Ryan R.M. (1985). Intrinsic Motivation and Self-Determination in Human Behavior.

[7] Appleton P.R., Hill A.P. (2012). Perfectionism and Athlete Burnout in Junior Elite Athletes: The Mediating Role of Motivation Regulations. J. Clin. Sport Psychol..

[8] Ntoumanis N., Edmunds J., Duda J.L. (2011). Understanding the Coping Process from a Self-Determination Theory Perspective. Br. J. Health Psychol..

[9] Mouratidis A., Michou A. (2011). Perfectionism, Self-Determined Motivation, and Coping among Adolescent Athletes. Psychol. Sport Exerc..

[10] Frost R.O., Marten P., Lahart C., Rosenblate R. (1990). The Dimensions of Perfectionism. Cogn. Ther. Res..

[11] Hewitt P.L., Flett G.L. (1991). Perfectionism in the Self and Social Contexts: Conceptualization, Assessment, and Association with Psychopathology. J. Personal. Soc. Psychol..

[12] Stoeber J., Otto K. (2006). Positive Conceptions of Perfectionism: Approaches, Evidence, Challenges. Personal. Soc. Psychol. Rev..

[13] Gotwals J.K., Dunn J.G.H. (2009). A Multi-Method Multi-Analytic Approach to Establishing Internal Construct Validity Evidence: The Sport Multidimensional Perfectionism Scale 2. Meas. Phys. Educ. Exerc. Sci..

[14] Dunkley D.M., Blankstein K.R., Halsall J., Williams M., Winkworth G. (2000). The Relation between Perfectionism and Distress: Hassles, Coping, and Perceived Social Support as Mediators and Moderators. J. Couns. Psychol..

[15] Gaudreau P., Thompson A. (2010). Testing a 2×2 Model of Dispositional Perfectionism. Personal. Individ. Differ..

[16] Stoeber J., Stoeber F.S. (2009). Domains of Perfectionism: Prevalence and Relationships with Perfectionism, Gender, Age, and Satisfaction with Life. Personal. Individ. Differ..

[17] Madigan D.J., Stoeber J., Passfield L. (2015). Perfectionism and Burnout in Junior Athletes: A Three-Month Longitudinal Study. J. Sport Exerc. Psychol..

[18] Květon P., Jelínek M., Burešová I. (2021). The Role of Perfectionism in Predicting Athlete Burnout, Training Distress, and Sports Performance: A Short-Term and Long-Term Longitudinal Perspective. J. Sports Sci..

[19] Hill A. (2013). Perfectionism and Burnout in Junior Soccer Players: A Test of the 2 × 2 Model of Dispositional Perfectionism. J. Sport Exerc. Psychol..

[20] Hill A.P., Curran T. (2016). Multidimensional Perfectionism and Burnout: A Meta-Analysis. Pers. Soc. Psychol. Rev..

[21] Hill A.P., Hall H.K., Appleton P.R., Kozub S.A. (2008). Perfectionism and Burnout in Junior Elite Soccer Players: The Mediating Influence of Unconditional Self-Acceptance. Psychol. Sport Exerc..

[22] Holmberg P. (2013). Self-Determined Motivation as a Predictor of Burnout Among College Athietes. Sport Psychol..

[23] Ryan R.M., Deci E.L. (2000). Self-Determination Theory and the Facilitation of Intrinsic Motivation, Social Development, and Well-Being. Am. Psychol..

[24] Cresswell S.L., Eklund R.C. (2005). Motivation and Burnout among Top Amateur Rugby Players. Med. Sci. Sports Exerc..

[25] Li C., Wang C.K.J., Pyun D.Y., Kee Y.H. (2013). Burnout and Its Relations with Basic Psychological Needs and Motivation among Athletes: A Systematic Review and Meta-Analysis. Psychol. Sport Exerc..

[26] Goodger K., Gorely T., Lavallee D., Harwood C. (2007). Burnout in Sport: A Systematic Review. Sport Psychol..

[27] Sarıkabak M., Sarı I., Kolayiş H., Toros T. Perfectionism and Motivation in Athletes: Negative Effect of Maladaptive Perfectionism on Motivation. Proceedings of the Prague International Conference 27.

[28] Barcza-Renner K., Eklund R.C., Morin A.J., Habeeb C.M. (2016). Controlling Coaching Behaviors and Athlete Burnout: Investigating the Mediating Roles of Perfectionism and Motivation. J. Sport Exerc. Psychol..

[29] Lazanis R.S. (1999). Stress and Emotion, a New Synthesis. J. Psychiatr. Ment. Health Nurs..

[30] Folkman S., Lazarus R.S. (1980). An Analysis of Coping in a Middle-Aged Community Sample. J. Health Soc. Behav..

[31] Shin H., Park Y.M., Ying J.Y., Kim B., Noh H., Lee S.M. (2014). Relationships between Coping Strategies and Burnout Symptoms: A Meta-Analytic Approach. Prof. Psychol. Res. Pract..

[32] Gaudreau P., Antl S. (2008). Athletes’ Broad Dimensions of Dispositional Perfectionism: Examining Changes in Life Satisfaction and the Mediating Role of Sport-Related Motivation and Coping. J. Sport Exerc. Psychol..

[33] Junior J.R.N., Oliveira L., Vissoci J.R.N., Ferreira L., Vieira L., Freire G., Silva A., de Moraes J.F., Vieira J. (2020). Perfectionism Traits and Coping Strategies in Soccer: A Study on Athletes’ Training Environment. J. Phys. Educ. Sport.

[34] Chang Y. (2012). The Relationship between Maladaptive Perfectionism with Burnout: Testing Mediating Effect of Emotion-Focused Coping. Personal. Individ. Differ..

[35] Li X., Hou Z.-J., Chi H.-Y., Liu J., Hager M.J. (2014). The Mediating Role of Coping in the Relationship between Subtypes of Perfectionism and Job Burnout: A Test of the 2×2 Model of Perfectionism with Employees in China. Personal. Individ. Differ..

[36] Pacewicz C.E., Gotwals J.K., Blanton J.E. (2018). Perfectionism, Coping, and Burnout among Intercollegiate Varsity Athletes: A Person-Oriented Investigation of Group Differences and Mediation. Psychol. Sport Exerc..

[37] Hill A.P., Hall H.K., Appleton P.R. (2010). Perfectionism and Athlete Burnout in Junior Elite Athletes: The Mediating Role of Coping Tendencies. Anxiety Stress Coping.

[38] Amiot C.E., Gaudreau P., Blanchard C.M. (2004). Self-Determination, Coping, and Goal Attainment in Sport. J. Sport Exerc. Psychol..

[39] Jowett G., Hill A., Hall H., Curran T. (2013). Perfectionism and Junior Athlete Burnout: The Mediating Role of Autonomous and Controlled Motivation. Sport Exerc. Perform. Psychol..

[40] Zhang L.W., Mao Z.X. (2004). Handbook of Psychological Scales for Sport Sciences.

[41] Zhou Y.G., Guo Y.J. (2007). Correlation between kinetic fatigue, social support and mental health with respect to excellent athletes. J. Phys. Educ..

[42] Lian W.J., Mao Z.X., Zi F. (2007). Measurement and application of perfectionism personality traits in Chinese college athletes. Int. J. Sport Exerc. Psychol..

[43] Dunn J.G.H., Dunn J.C., Syrotuik D.G. (2002). Relationship between Multidimensional Perfectionism and Goal Orientations in Sport. J. Sport Exerc. Psychol..

[44] Ding Y., Lu G., Chen S., Liang Y., Zhang Y., Peng Q., Liang S., Chen C. (2023). The Effect of Perfectionism on Relative Deprivation among Nursing Students: The Role of Interpersonal Sensitivity and Resilience. Psychol. Sch..

[45] Guay F., Vallerand R.J., Blanchard C. (2000). On the Assessment of Situational Intrinsic and Extrinsic Motivation: The Situational Motivation Scale (SIMS). Motiv. Emot..

[46] Zhong B.G., Si G.Y., Li Q.Z., Liu H. (2004). Development and Preliminary Test of Coping Scale for Chinese Athletes. Chin. J. Sports Med..

[47] Tabachnick B.G., Fidell L.S. (2007). Using Multivariate Statistics.

[48] Hayes A. (2013). Introduction to Mediation, Moderation, and Conditional Process Analysis. J. Educ. Meas..

[49] Cohen J. (1988). Statistical Power Analysis for the Behavioral Sciences.

[50] Gnilka P., McLaulin S., Ashby J., Allen M. (2017). Coping Resources As Mediators Of Multidimensional Perfectionism And Burnout. Consult. Psychol. J. Pract. Res..

[51] Einstein D.A., Lovibond P.F., Gaston J.E. (2000). Relationship between Perfectionism and Emotional Symptoms in an Adolescent Sample. Aust. J. Psychol..

[52] Rozental A. (2020). Beyond Perfect? A Case Illustration of Working with Perfectionism Using Cognitive Behavior Therapy. J. Clin. Psychol..

[53] Lasalle M., Hess U. (2022). A Motivational Approach to Perfectionism and Striving for Excellence: Development of a New Continuum-Based Scale for Post-Secondary Students. Front. Psychol..

[54] Sheehan R.B., Herring M.P., Campbell M.J. (2018). Associations Between Motivation and Mental Health in Sport: A Test of the Hierarchical Model of Intrinsic and Extrinsic Motivation. Front. Psychol..

[55] Miquelon P., Vallerand R.J., Grouzet F.M.E., Cardinal G. (2005). Perfectionism, Academic Motivation, and Psychological Adjustment: An Integrative Model. Pers. Soc. Psychol. Bull..

[56] Atienza F.L., Castillo I., Appleton P.R., Balaguer I. (2020). Examining the Mediating Role of Motivation in the Relationship between Multidimensional Perfectionism and Well- and Ill-Being in Vocational Dancers. Int. J. Environ. Res. Public Health.

[57] Madigan D.J., Rumbold J.L., Gerber M., Nicholls A.R. (2020). Coping Tendencies and Changes in Athlete Burnout over Time. Psychol. Sport Exerc..

[58] Wiese-Bjornstal D.M. (2010). Psychology and Socioculture Affect Injury Risk, Response, and Recovery in High-intensity Athletes: A Consensus Statement. Scand. Med. Sci. Sports.

[59] Ogoma S. (2020). Problem-Focused Coping Controls Burnout in Medical Students: The Case of a Selected Medical School in Kenya. J. Psychol. Behav. Sci..

[60] Maxwell S.E., Cole D.A., Mitchell M.A. (2011). Bias in Cross-Sectional Analyses of Longitudinal Mediation: Partial and Complete Mediation under an Autoregressive Model. Multivar. Behav. Res..

